# Fungal Volatiles Can Act as Carbon Sources and Semiochemicals to Mediate Interspecific Interactions Among Bark Beetle-Associated Fungal Symbionts

**DOI:** 10.1371/journal.pone.0162197

**Published:** 2016-09-01

**Authors:** Jonathan A. Cale, R. Maxwell Collignon, Jennifer G. Klutsch, Sanat S. Kanekar, Altaf Hussain, Nadir Erbilgin

**Affiliations:** 1 Department of Renewable Resources, 4-42 Earth Sciences Building, University of Alberta, Edmonton, Alberta, T6G 2E3, Canada; 2 Department of Entomology, Entomology Building, University of California, Riverside, CA, 92521, United States of America; Swedish University of Agricultural Sciences, SWEDEN

## Abstract

Mountain pine beetle (*Dendroctonus ponderosae*) has killed millions of hectares of pine forests in western North America. Beetle success is dependent upon a community of symbiotic fungi comprised of *Grosmannia clavigera*, *Ophiostoma montium*, and *Leptographium longiclavatum*. Factors regulating the dynamics of this community during pine infection are largely unknown. However, fungal volatile organic compounds (FVOCs) help shape fungal interactions in model and agricultural systems and thus may be important drivers of interactions among bark beetle-associated fungi. We investigated whether FVOCs can mediate interspecific interactions among mountain pine beetle’s fungal symbionts by affecting fungal growth and reproduction. Headspace volatiles were collected and identified to determine species-specific volatile profiles. Interspecific effects of volatiles on fungal growth and conidia production were assessed by pairing physically-separated fungal cultures grown either on a carbon-poor or -rich substrate, inside a shared-headspace environment. Fungal VOC profiles differed by species and influenced the growth and/or conidia production of the other species. Further, our results showed that FVOCs can be used as carbon sources for fungi developing on carbon-poor substrates. This is the first report demonstrating that FVOCs can drive interactions among bark beetle fungal symbionts, and thus are important factors in beetle attack success.

## Introduction

Bark beetles (Coleoptera: Curculionidae, Scolityinae) are among the most destructive tree-killing insects in temperate and boreal conifer forests worldwide. In general, bark beetles remain at low densities for decades—suppressed by competitors, natural enemies, and host tree defenses—and are restricted to hosts with weakened defenses [1], which are typically rare in the landscape. However, under more favorable environmental conditions, beetle populations increase and enter an outbreak phase, during which host mortality may approach near 100%, potentially over millions of hectares [2–4]. In western North America, the native mountain pine beetle (*Dendroctonus ponderosae* Hopkins; MPB) has killed at least 28 million hectares of primarily lodgepole pine (*Pinus contorta* Douglas ex Loudon) forests during the last outbreak [4–7]. This outbreak has altered forest structure and succession as well as below- and aboveground species diversity [5,8–10]. Importantly, successful MPB attack and survival relies upon the activities of the beetle’s symbiotic fungal community [11,12].

Mountain pine beetle vectors many microorganisms including bacteria, mites, yeasts, and filamentous fungi [13–16]. In particular, three phytopathogenic, ophiostomatoid fungi (Ascomycota: Ophiostomataceae) are critical to beetle attack and larval development in Canadian forests: *Grosmannia clavigera* (Robinson-Jeffrey and Davidson) Zipfel, de Beer, and Wing., *Ophiostoma montium* (Rumford) von Arx, and *Leptographium longiclavatum* Lee, Kim, and Breuil [17–20]. Attacking beetles transfer primarily asexual fungal spores (conidia) from their mycangia—specialized structures for transporting fungal propagules—to pine phloem [12,15]. Fungal infections in pine phloem and functional xylem (sapwood) weaken host trees, further making these trees less resistant to MPB attacks [2,10]. Fungi are an important food source for developing beetles as pine phloem is nutrient-poor and hyphae are a source of nitrogen for beetle larvae [21,22]. Further, fungal ergosterol is an important nutrient factor for beetle metamorphosis and reproduction [23]. Fungi colonize and sporulate within beetle pupal chambers, which is critical to fungal dispersal and population growth as emerging adult beetles feed on and fill their mycangia with conidia before seeking new host trees [11,15]. The overall benefits these fungi impart to MPB, and in turn the impacts of beetles on forests, vary by species and thus are a function of fungal community composition [11]. This composition is in part determined by competitive exclusion and co-existence [15,20], however the mechanisms regulating interactions among bark beetle-associated fungi are unknown.

While largely unexplored, volatile organic compounds emitted by fungi (FVOCs) are receiving increasing recognition for their importance in mediating many aspects of fungal ecology [24–27]. Fungal VOCs can represent several classes of bioactive chemicals, such as acids, alcohols, aldehydes, esters, ketones, terpenes, and thiols [25,26]. These compounds are produced by fungi occupying many ecological niches (e.g., saprophytes, symbionts, and parasites), and influence how fungi interact with plants, animals, and other fungi [24,28–32]. Indeed, many interactions among fungi are dependent upon FVOCs. For example, as developmental signals during population establishment, certain FVOCs act in a concentration-dependent manner to regulate conspecific mycelial growth and spore germination [33–35]. These compounds can additionally function to regulate mycelial growth or asexual spore production of other fungi [36,37]. By eliciting inhibitory or stimulatory responses from other species, FVOCs can drive antagonistic or beneficial interactions among fungi [38,39]. In these interactions, fungal VOCs are often described to function as semiochemicals—information signals or cues that elicit behavioral responses from recipients. However, whether FVOCs affect other aspects of fungal development for instance by serving as carbon sources, as has been shown for many industrial VOCs [40,41], is unknown, but could allow co-occurring fungi access to different carbon pool and thus help explain resource partitioning in fungal communities. Although our understanding of FVOC ecology is primarily from model or agricultural systems, these compounds likely play critical and potentially novel ecological roles in natural systems [24,42]. While ophiostomatoid fungi produce a wide variety of VOCs [43], the potential importance of these chemicals in regulating communities of bark beetle fungal symbionts is unknown.

Here, the MPB symbionts *G*. *clavigera*, *O*. *montium*, and *L*. *longiclavatum* were used in laboratory experiments to investigate the potential importance of FVOCs in regulating interactions among bark beetle fungal symbionts. We used headspace volatile collections as well as paired-growth experiments on carbon-poor and -rich substrates in shared-headspace environments to pursue several research questions. (1) Can FVOC profiles of these fungi qualitatively and/or quantitatively differ from each other? (2) Can the VOCs emitted by one fungus affect the growth and spore production of other species? (3) Can these fungi use FVOCs from other species as a carbon source? We demonstrate that the mode by which different FVOC profiles effect fungi can be context dependent. Specifically, these compounds are likely important carbon sources for fungi colonizing carbon-limited substrates. Conversely, for fungi colonizing more carbon-rich substrates, FVOCs may act, in a concentration-dependent manner, as semiochemicals to mediate antagonistic and beneficial interactions between fungi.

## Materials and Methods

### Collection and quantification of fungal volatiles

A push-pull system was designed to sample headspace volatiles from cultures of the MPB-associated symbionts, *G*. *clavigera*, *O*. *montium*, and *L*. *longiclavatum*, as well as non-inoculated controls of potato dextrose agar (24 g potato dextrose broth, 15 g agar, and 1 L distilled water; PDA). Fungal cultures were obtained from different sources: *G*. *clavigera* was originally isolated from MPB in Fox Creek, Alberta and provided by AV Rice (Northern Forestry Centre, Canadian Forest Service, Edmonton, Alberta), *L*. *longiclavatum* (NOF 3100) was provided by the Northern Forestry Centre Culture Collection, and *O*. *montium* (UAMH 4838) was provided by the University of Alberta Microfungus Collection and Herbarium (Edmonton). These strains had morphologies and growth rates comparable to others in our collection and were thus considered representative of their species. Fungal culture and control plates were each replicated 15 times. Cultures were grown by subculturing (using 5 mm diameter plugs) the actively growing margin of eight-day old cultures onto small PDA plates (60 cm x 15 cm). These subcultures were grown in permanent darkness at 22°C for four days. After this period, culture margins were traced, photographed, and used to quantify culture area using ImageJ software (National Institutes of Health, Bethesda, MD, USA) [44]. Cultures were then placed into a volatile collection chamber consisting of a 473 mL glass jar with Teflon tape on its threading and fitted with a metal cap. Two holes were drilled in the caps, fitting a Teflon tube (6.35 mm od) through each. Activated carbon, (800 mg; 6–14 mesh, Fisher Scientific, Hampton, NH, USA), fixed in place with glass wool ends, was packed halfway down the first tube to filter incoming ambient air. This inlet tube was attached to a metal gang-valve connected to the outlet spigot of a bellows vacuum/pressure pump (Cole-Parmer Canada Inc., Montreal, QC, Canada; #UZ-79600-04). The second tube was attached to a volatile trap consisting of 150 mg of the activated carbon, held in place by glass wool ends, within a 7.5 cm piece of Teflon tube (4.75 mm od). Another tube joined this trap to a gang-valve connected to the pump inlet spigot. Constant flow through chamber lines was set to 450 mL min^-1^ using a flowmeter. Each gang-valve manifold was connected to four volatile collection chambers. One culture or control was placed into each chamber, with Petri plate lids set ajar approximately 5 mm to encourage volatile diffusion. Cultures were sealed in separate chambers such that there was one jar containing a culture of each fungus and a control attached to a given gang-valve. Headspace volatiles were then collected for 24 h after which time the carbon traps were removed from the collection apparatus and extracted.

Volatiles were extracted by adding the activated carbon to a microtube containing 1 mL of dichloromethane containing a tridecane internal standard (0.002%). This mixture was vortexed for 30 sec, sonicated for 10 min, and centrifuged (at 30,000 rpm) for 30 min before the extract was collected and transferred to a gas chromatograph (GC) vial. This procedure was repeated a second time before chromatographic separation. Extracts were analyzed using a GC fitted with a DB-5MS UI column (30 m x 0.25 mm ID x 0.25 μm film, product: 122-5532UI; Agilent Tech, Santa Clara, CA, USA) and coupled to a mass spectrometer (GC-MS; GC: 7890A, MS: 5062C, Agilent Tech., Santa Clara, CA, USA). Helium was used as a carrier gas flowing at 1 mL min^-1^ with a temperature program beginning at 50°C (held for 1 min) then increased by 5°C min^-1^ to 200°C, followed by an increase of 30°C min^-1^ to 325°C (held for 2 min). A 1 μl sample injection volume was used, the injector temperature was 250°C, and samples were run in splitless mode. Peaks present in chromatographs of controls were ignored from those of fungal cultures to determine peaks unique to the latter sample groups. Library matches using NIST/EPA/NIH Mass Spectral library version 2.0f for all detected fungal volatiles were verified and quantified using the following standards: acetoin (≥ 96%), ethyl acetate (≥ 99%), *cis*-grandisol (≥ 96%), isoamyl acetate (≥ 97%), isobutanol (≥ 99%), 2-methyl-1-butanol (≥ 99% pure), 3-methyl-1-butanol (98%), phenethyl acetate (≥ 98%), and phenethyl alcohol (≥ 99%). All standards were purchased from Sigma-Aldrich (St. Louis, MO, USA), except *cis*-grandisol which was purchased from Alpha Scents (West Linn, OR, USA). Analyte concentrations were standardized by culture area prior to data analysis.

### Volatile-mediated interactions

Potential volatile-mediated interactions between pairings of *G*. *clavigera*, *O*. *montium*, and *L*. *longiclavatum* were investigated in cross-experiments testing concurrent or staggered growth between fungi. The concurrent-growth experiment investigated interactions between freshly inoculated (young) cultures. Young cultures of each fungus were subcultured (using 5 mm diameter plugs) onto small PDA plates from actively growing margins of eight-day old cultures. Subculture plates were immediately placed into a volatile exposure chamber consisting of a 473 mL glass jar whose threading was wrapped in Teflon tape and fitted with a metal cap. Placed in the chamber was a shelf made from a piece of steel wire whose ends were coiled and bent along the horizontal plane so they were parallel to one another and connected by a 2.5 cm straight section of wire. A culture plate of one fungus was placed onto the bottom of the chamber while the wire shelf held a plate of a different fungus above. Culture plate lids were set ajar approximately 5 mm to encourage FVOC diffusion within the sealed chambers. This setup allowed us to examine volatile-mediated interactions in a shared-headspace environment while preventing all physical contact between fungi. Interspecific interactions were investigated between fungi in three pairing treatments: *G*. *clavigera*-*O*. *montium*, *G*. *clavigera*-*L*. *longiclavatum*, *O*. *montium*-*L*. *longiclavatum*. Fungus-non-inoculated PDA pairs were used as controls for each species. Treatment pairings and controls were each replicated 20 times. Potential intraspecific, volatile-mediated interactions were not investigated because we used only one strain of each fungus. Vertical placement of cultures (or controls) within chambers was evenly divided among replicates. For example, in the first ten replicates *G*. *clavigera* cultures were placed on the shelf above *O*. *montium* cultures, while the opposite placement was used in the last ten replications. This allowed us to examine potential effects of vertical placement on inter-fungal interactions of fungal characteristics. No significant effects were detected (p>0.05 for each experiment). Chambers with treatment and control pairings were placed in permanent darkness at 22°C for three days.

Cultures were then removed from the chambers, culture area was quantified as described above, and conidia production was determined from a 1 mm tall section of the inoculation plug (5 mm diameter) used to originally inoculate the plates. This section was used for three reasons: (1) sporulation often occurs on older parts of fungal cultures before younger parts; (2) cultures had a short growth period which likely did not allow more distal hyphal sections to mature and sporulate before being removed from the chamber; and (3) conidia and conidiophores were not observed on any other part of the culture during preliminary examinations. Further, preliminary comparisons indicated conidia density estimates from the inoculation plug section were not significantly different (p>0.05; n = 15 for each fungus) for any of the fungi from estimates made by flooding culture plates. Conidia production was quantified by vortexing the 1 mm (5 mm diameter) section in a microtube with 1 mL 0.5% Tween20 for 30 sec. An aliquot of this spore suspension was pipetted into a hemocytometer, which was used to quantify conidia concentration (number per mL). Conidia concentrations were standardized using culture area (plus the plug section area) prior to data analysis.

The staggered-growth experiment investigated the effects of volatiles from older (four-day old) cultures on the growth and conidia production of younger (i.e., freshly inoculated) cultures. Old cultures were inoculated onto PDA and grown at 22°C in permanent darkness for four days after which they were placed into and sealed within volatile exposure chambers with young, newly-inoculated PDA cultures of a different fungus. Vertical placement of cultures was stratified as described in the concurrent-growth experiment above. Six old-young pairings were investigated: old *G*. *clavigera*-young *O*. *montium*, old *G*. *clavigera*-young *L*. *longiclavatum*, old *O*. *montium*-young *G*. *clavigera*, old *O*. *montium*-young *L*. *longiclavatum*, old *L*. *longiclavatum*-young *G*. *clavigera*, and old *L*. *longiclavatum*-young *O*. *montium*. Controls were prepared for each fungus by placing a young culture in a chamber with a non-inoculated PDA plate. Pairings and controls were each replicated 20 times. Chambers were sealed and placed in permanent darkness at 22°C for three days. After this period, young cultures were removed, and culture area and conidia production were quantified as described above.

### Carbon-restricted growth

Volatile exposure chambers were further used to investigate whether FVOCs could serve as carbon sources for MPB symbiotic fungi developing on substrates without usable carbon. This experiment was identical in design to the staggered-growth experiment above, except young cultures were prepared by subculturing master cultures grown on yeast nitrogen base agar (YNB; Fisher Scientific, Hampton, NH, USA) onto freshly prepared YNB agar plates. This media was chosen because it lacks carbohydrates but contains minerals essential for fungal growth. Therefore, inoculation plugs and plate media were devoid of usable carbon. Similarly, agar is not known to be digested by ophiostomatoid fungi and thus is not a carbon source. Yeast nutrient base agar was prepared by first making a 1.7% YNB solution (2.55 g YNB in 150 mL distilled water) and a 2.4% water-agar solution (20 g agar in 850 mL distilled water). The latter was autoclaved and set to cool slightly at which time the YNB solution was filter sterilized (Millex-GS 0.22 μm filter, Merck EMD Millipore Ltd., Billerica, MA, USA) and added to the agar before pouring plates. The area of YNB agar cultures were measured after a three-day growth period during which time chambers were kept at 22°C in permanent darkness. Usable carbon content of FVOC profiles was inferred from the area of YNB agar cultures instead of analytically quantifying mycelial carbon content because the biomass of these cultures was below the minimum for such analytical analysis. This experiment is based on preliminary work using a similar design that used master cultures grown on potato dextrose agar and a water-agar plate media for subculture growth. The results of this experiment are presented as Supporting Information (S1 Fig).

### Data analysis

Descriptive statistics were calculated for culture area (mm^2^), FVOC concentrations (μg mm^-2^ of culture), and conidia density (conidia mm^-2^ of culture). Quantitative differences in FVOC profiles among fungi as well as identification of FVOCs most associated with each fungus were examined by permutational MANOVA (PerMANOVA) and non-metric multidimensional scaling (NMDS). Family-wise differences in mean FVOC concentrations among fungi as well as culture area and conidia density among treatments and controls of the interaction experiments were examined for statistical significance by ANOVA. Following significant ANOVA results, pair-wise differences were tested using Tukey Honest Significant Difference multiple comparison tests. Data were natural-log or rank transformed to satisfy statistical assumptions of normality and heteroscedasticity, as necessary. Raw, non-transformed data were reported in tables and used to construct figures. All statistical analyses were performed using the R software environment version 3.2.1. [45]. The PerMANOVA and NMDS analyses were performed using functions provided in R package “vegan” version 2.3–2 [46]. All research data are publically available online (doi: 10.7939/DVN/10689).

## Results

### Volatile profiles

**Fig 1:**
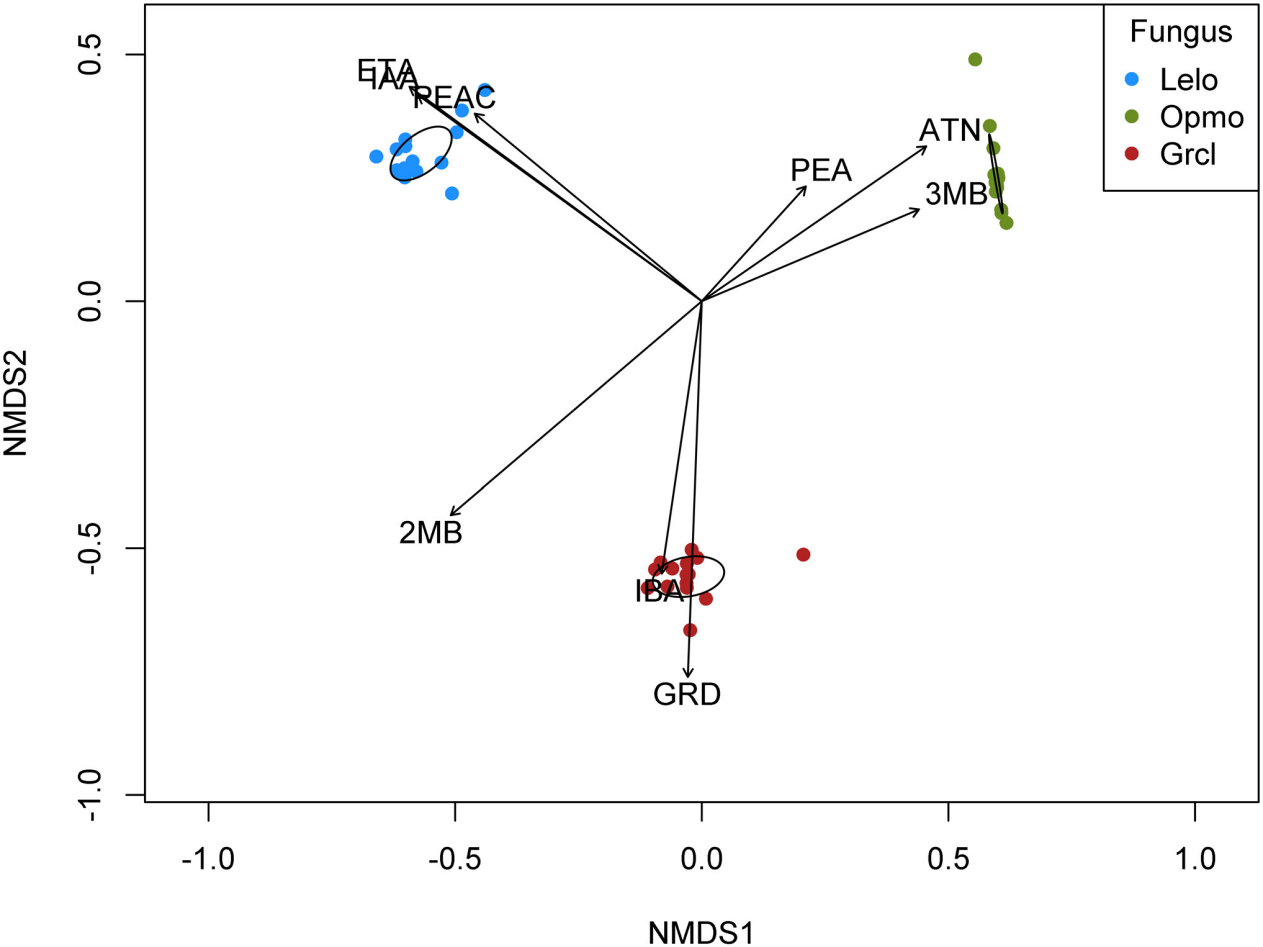
NMDS results showing the separation of *Ophiostoma montium* (Opmo), *Leptographium longiclavatum* (Lelo), and *Grosmannia clavigera* (Grcl) based on concentrations (ng mm^-2^) of nine headspace volatiles: acetoin (ATN), ethyl acetate (ETA), *cis*-grandisol (GRD), isobutanol (IBA), isoamyl acetate (IAA), 2-methyl-1-butanol (2MB), 3-methyl-1-butanol (3MB), phenethyl acetate (PEAC), and phenethyl alcohol (PEA). Black ellipses indicate 95% confidence intervals around cluster centroids.

**Fig 2:**
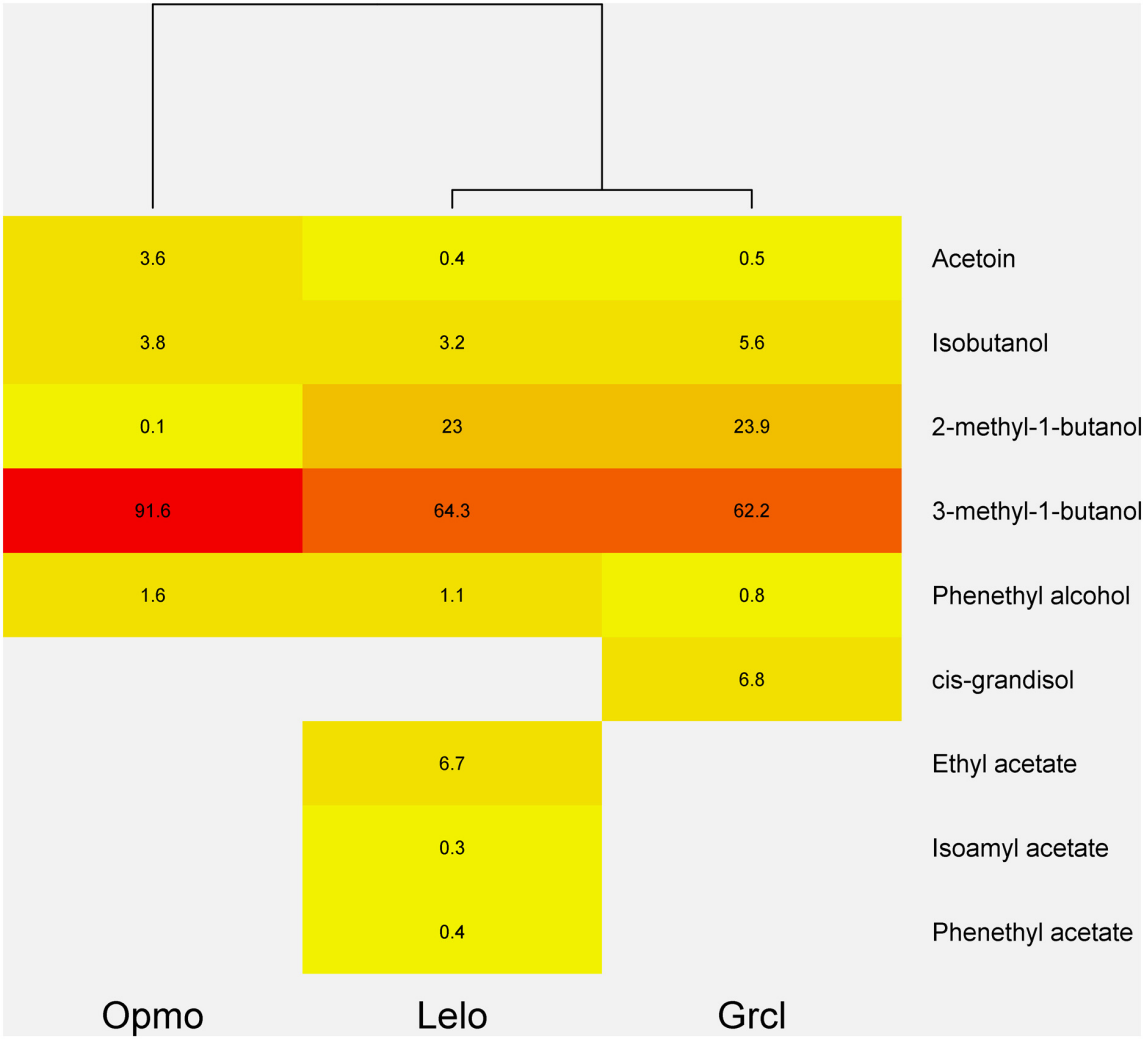
The composition of headspace volatile (rows) profiles from four-day old *Ophiostom montium* (Opmo), *Leptographium longiclavatum* (Lelo), and *Grosmannia clavigera* (Grcl) cultures. Cells contain the percentage of each volatile out of the total concentration of all detected volatiles by fungus. The dendrogram shows similarities among fungi based on volatile profile compositions as indicated by hierarchical clustering.

Nine FVOCs, representing three classes of carbon-based chemicals, were detected in extractions of headspace volatiles of *G*. *clavigera*, *O*. *montium*, and *L*. *longiclavatum* adsorbed to activated carbon during a 24 h period: acetoin (ketone), ethyl acetate isoamyl acetate, and phenethyl acetate (esters), *cis*-grandisol (also known as grandlure I), isobutanol, 2-methyl-1-butanol, 3-methyl-1-butanol, and phenethyl alcohol (alcohols). Fungal VOC profiles significantly differed among *G*. *clavigera*, *O*. *montium*, and *L*. *longiclavatum* (PerMANOVA *F*_2, 42_ = 31.16, p<0.001). Indeed, NMDS indicated clear separation among fungi and tight within-species clustering based on FVOC profiles (Fig 1). Further, NMDS showed that *G*. *clagivera* was most closely associated with *cis*-grandisol and isobutanol, *O*. *montium* was associated with acetoin and 3-methyl-1-butanol, and *L*. *longiclavatum* was associated with ethyl acetate, isoamyl acetate, and phenethyl acetate (Fig 1). *Leptographium longiclavatum* showed the richest FVOC profile, emitting eight of nine detected compounds (Table 1). Further, *L*. *longiclavatum* and *G*. *clavigera* emitted exclusive compounds: *cis*-grandisol being exclusive to the latter, while ethyl acetate, isoamyl acetate, and phenethyl acetate were exclusive to the former (Table 1). We detected several significant differences in mean concentrations of individual FVOCs among and between fungi (Table 1). Fungal VOC profile composition varied among fungi (Fig 2). While most individual compounds accounted for less than 10%, 3-methyl-1-butanol accounted for at least 62.2% of total FVOC concentrations from each fungus (Fig 2). *Ophiostoma montium* showed a composition distinct from those of *G*. *clavigera* and *L*. *longiclavatum*, which were similar to each other (Fig 2).

**Table 1:** Mean concentrations (ng mm^-2^) of detected headspace volatiles from four-day old cultures of mountain pine beetle (*Dendroctonus ponderosae*)-symbiotic fungi: *Grosmannia clavigera*, *Ophiostoma montium*, and *Leptographium longiclavatum*. ANOVA results for among-species differences in mean concentrations for each compound. Means with different letter superscripts are significantly different as indicated by Tukey Honest Significant Difference tests. Compounds not detected in headspace collections of a given fungus are indicated with “ND.”

	Mean (±s.e.) concentration (ng mm^-2^)			ANOVA	
Compound	*G*. *clavigera*	*O*. *montium*	*L*. *longiclavatum*	*F*_2, 42_	p-value
Acetoin	0.91 (±0.15)^a^	5.87 (±1.17)^b^	0.54 (±0.07)^c^	53.31	<0.001
Isobutanol	8.64 (±0.58)^a^	4.80 (±0.33)^b^	5.44 (±0.29)^b^	10.21	<0.001
2-methyl-1-butanol	40.41 (±4.26)^a^	0.07 (±3.23)^b^	33.69 (±3.23)^a^	8.59	<0.001
3-methyl-1-butanol	103.22 (±7.81)^a^	139.27 (±8.53)^b^	91.09 (±4.17)^a^	10.43	<0.001
Phenethyl alcohol	1.53 (±0.26)^a^	2.70 (±0.61)^b^	1.55 (±0.22)^ab^	12.47	0.043
*cis*-grandisol	10.59(±0.31)	ND	ND	-	-
Ethyl acetate	ND	ND	9.46 (±0.74)	-	-
Isoamyl acetate	ND	ND	0.45 (±0.05)	-	-
Phenethyl acetate	ND	ND	0.52 (±0.09)	-	-

### Volatile-mediated interactions between fungi

**Fig 3:**
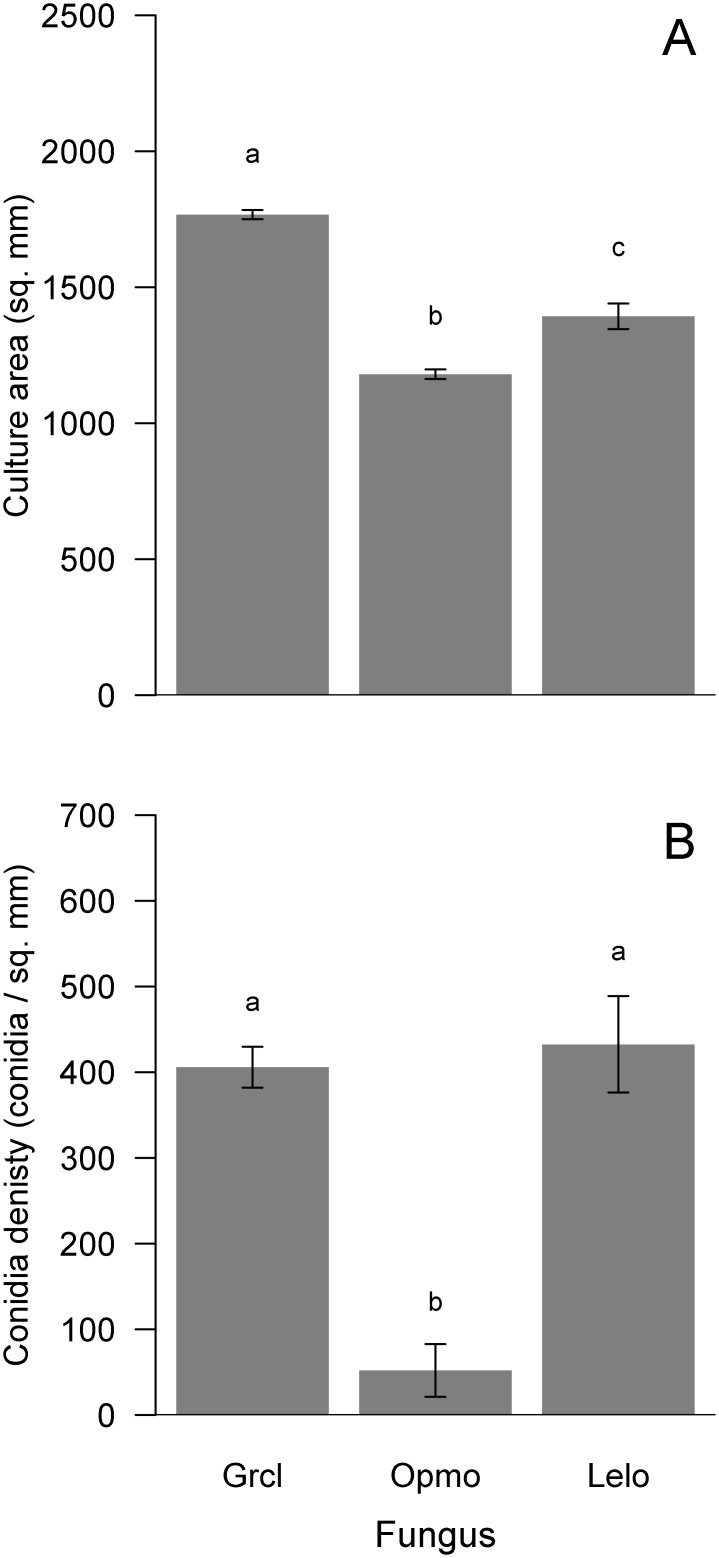
Mean (± s.e.) differences in culture area (mm^2^, A) and conidia density (conidia per mm^2^; B) among fungal symbionts, *Grosmannia clavigera* (Grcl), *Ophiostoma montium* (Opmo), and *Leptographium longiclavatum* (Lelo), of mountain pine beetle (*Dendroctonus ponderosae*) for controls of the concurrent-growth experiment. Bars with different letters are statistically different as indicated by Tukey Honest Significant Difference tests.

For the concurrent-growth experiment, we detected differences in culture area and conidia density among the control cultures of each fungus. Culture area of controls significantly differed among fungi (*F*_2, 57_ = 75.13, p<0.001; Fig 3A). *Grosmannia clavigera* controls had the largest area, being 33% and 21% larger than those of *O*. *montium* and *L*. *longiclavatum*, respectively (Fig 3A). Similarly, conidia density of control cultures also significantly differed among fungi (*F*_2, 57_ = 176.90, p<0.001; Fig 3B). Although conidia density did not differ between *G*. *clavigera* and *L*. *longiclavatum*, *O*. *montium* produced 87% and 88% fewer conidia than these fungi, respectively (Fig 3B).

**Fig 4:**
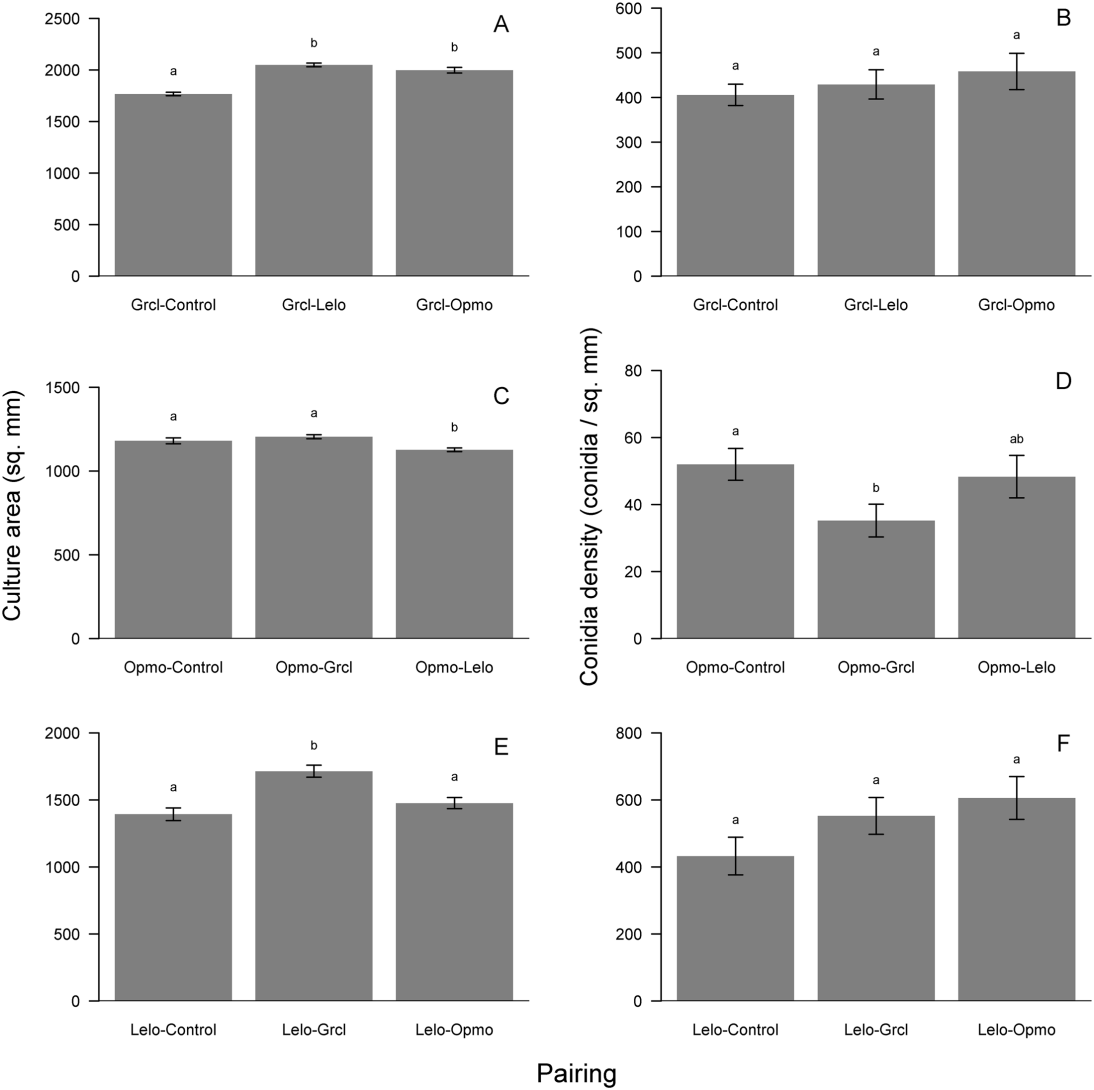
Results of concurrent-growth experiment showing mean (± s.e.) growth (mm^2^; left panels) and conidia density (conidia mm^-2^; right panels) responses to headspace volatiles of the other fungi in species-pairing treatments and non-inoculated agar controls for *Grosmannia clavigera* (Grcl; A, B), *Ophiostoma montium* (Opmo, C, D), and *Leptographium longiclavatum* (Lelo; E, F). Bars with different letters are statistically different as indicated by Tukey Honest Significant Difference tests.

Growth and conidia production responses of young cultures to FVOCs of their paired fungus differed by treatment. *Grosmannia clavigera* growth was stimulated by 16% and 13% when exposed to *L*. *longiclavatum* and *O*. *montium* FOVCs, respectively (*F*_2, 57_ = 60.18, p<0.001; Fig 4A). Further, *G*. *clavigera* conidia production did not respond to pairing treatments (Fig 4B). For *O*. *montium*, growth significantly varied among controls and treatment pairings, and was slightly (5%) inhibited by FVOCs from *L*. *longiclavatum* (*F*_2, 57_ = 8.16, p<0.001; Fig 4C). However, conidia production exhibited a stronger response to pairing treatments as *O*. *montium* significantly produced 32% fewer conidia when exposed to *G*. *clavigera* FVOCs (*F*_2, 57_ = 4.91, p = 0.004; Fig 4d). *Leptographium longiclavatum* growth significantly differed among controls and pairing treatments, and was stimulated by 23% when exposed to FOVCs of *G*. *clavigera* (*F*_2, 57_ = 14.98, p<0.001; Fig 4E). However, *L*. *longiclavatum* conidia production did not respond to pairing treatments (Fig 4F).

**Fig 5:**
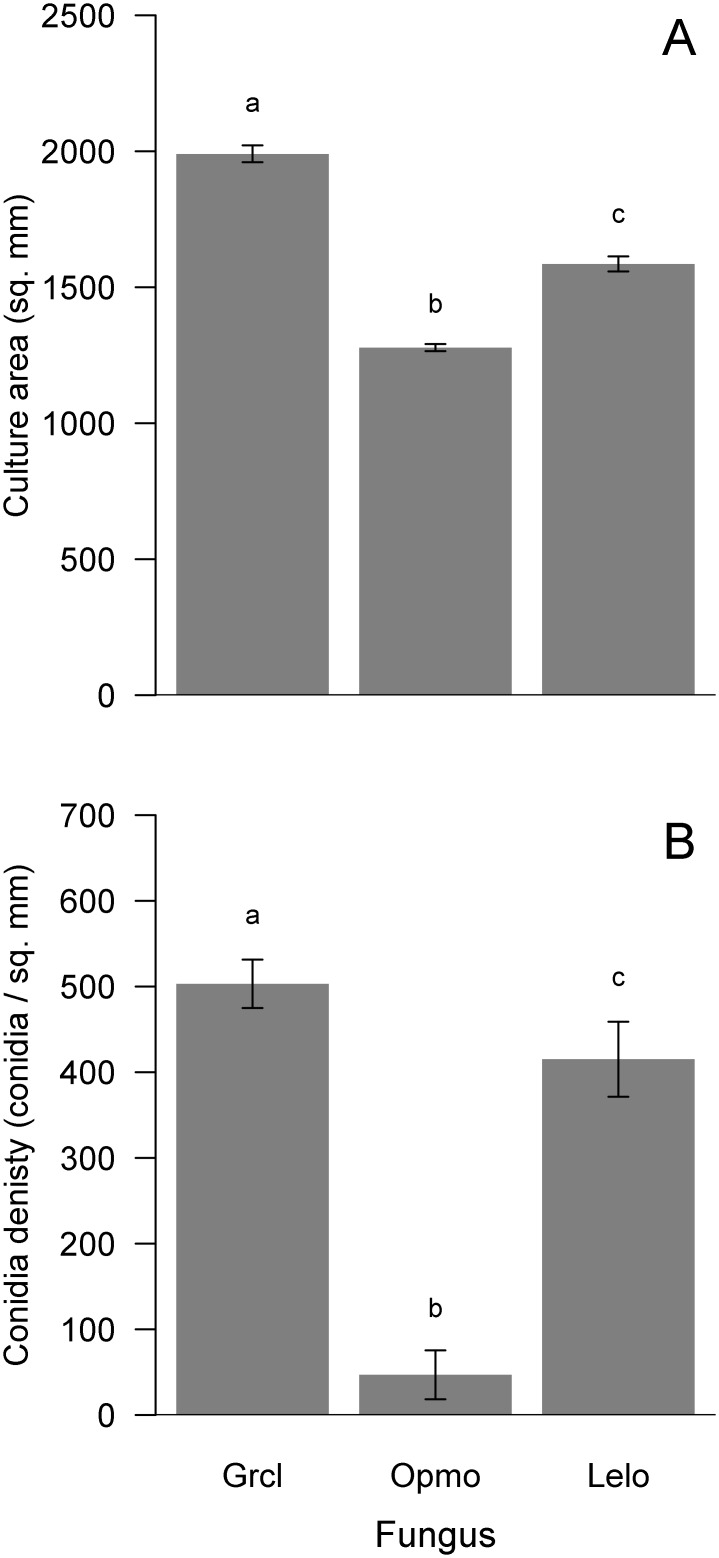
Mean (± s.e.) differences in culture area (mm^2^, A) and conidia density (conidia per mm^2^; B) among fungal symbionts, *Grosmannia clavigera* (Grcl), *Ophiostoma montium* (Opmo), and *Leptographium longiclavatum* (Lelo), of mountain pine beetle (*Dendroctonus ponderosae*) for controls of the staggered-growth experiment. Bars with different letters are statistically different as indicated by Tukey Honest Significant Difference tests.

For the staggered-growth experiment, the relative area and conidia density of control cultures were similar to those observed in the concurrent-growth experiment above. Culture area significantly varied among fungi, and *G*. *clavigera* cultures were 36% and 20% larger than *O*. *montium* and *L*. *longiclavatum* cultures, respectively (*F*_2, 57_ = 234.4, p<0.001; Fig 5A). Conidia density significantly differed among fungi (*F*_2, 57_ = 238.10, p<0.001; Fig 5B). *Grosmannia clavigera* produced on average 91% and 17% more conidia than *O*. *montium* and *L*. *longiclavatum*, respectively (Fig 5B).

**Fig 6:**
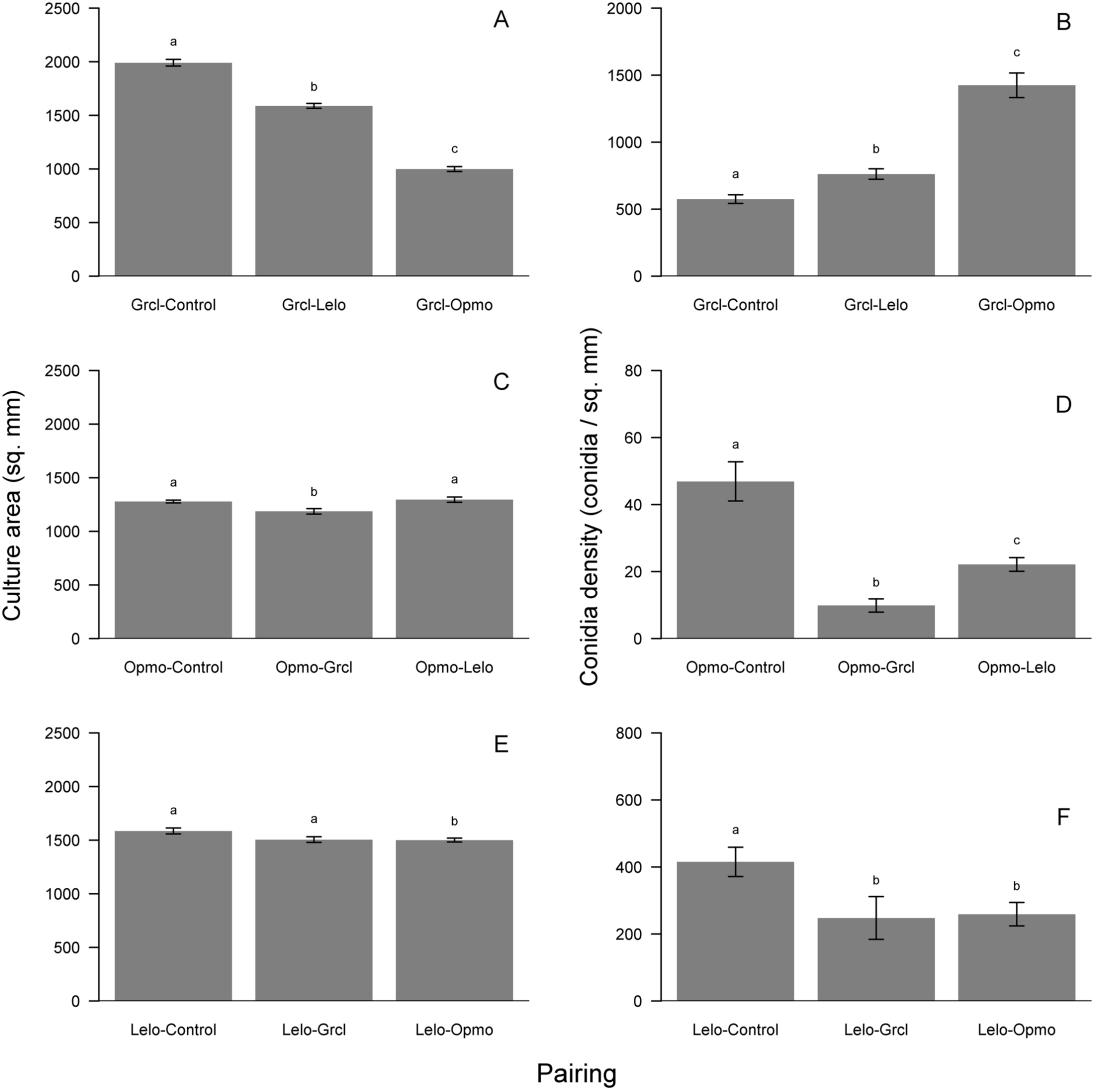
Results of staggered-growth experiment showing mean (± s.e.) growth (mm^2^; left panels) and conidia density (conidia mm^-2^; right panels) responses to headspace volatiles of the other fungi in species-pairing treatments and non-inoculated agar controls for *Grosmannia clavigera* (Grcl; A, B), *Ophiostoma montium* (Opmo, C, D), and *Leptographium longiclavatum* (Lelo; E, F). Bars with different letters are statistically different as indicated by Tukey Honest Significant Difference tests.

Fungal VOCs of old cultures differentially affected growth and conidia production of young cultures. *Grosmannia clavigera* showed significant growth reductions of 50% and 20% when exposed to FVOCs of old *O*. *montium* and *L*. *longiclavatum* cultures (*F*_2, 57_ = 373.80, p<0.001, Fig 6A), respectively. Volatiles from these fungi stimulated *G*. *clavigera* conidia production by 148% and 33%, respectively (*F*_2, 57_ = 52.23, p<0.001, Fig 6C). However, this inverse pattern was not observed for either other fungus, whose growth and conidia production were primarily inhibited by FVOCs from old cultures. For *O*. *montium*, culture growth (*F*_2, 57_ = 6.65, p = 0.003, Fig 6C) and conidia production (*F*_2, 57_ = 51.56, p<0.001, Fig 6D) significantly responded to pairing treatments. Cultures showed a 7% reduction in growth in the *G*. *clavigera* treatment (Fig 6C), but produced 79% and 53% fewer spores in the *G*. *clavigera* and *L*. *longiclavatum* treatments (Fig 6D), respectively, compared to controls. *Leptographium longiclavatum* showed significantly reduced growth (*F*_2, 57_ = 3.79, p = 0.029, Fig 6E) and conidia production (*F*_2, 57_ = 9.94, p<0.001, Fig 6F) in response to treatments. Specifically, growth was reduced by 5% for both *G*. *clavigera* and *O*. *montium* treatments (Fig 6E) while conidia production was reduced by 40% and 38% in these treatments (Fig 6F), respectively.

### Carbon-restricted growth

**Fig 7:**
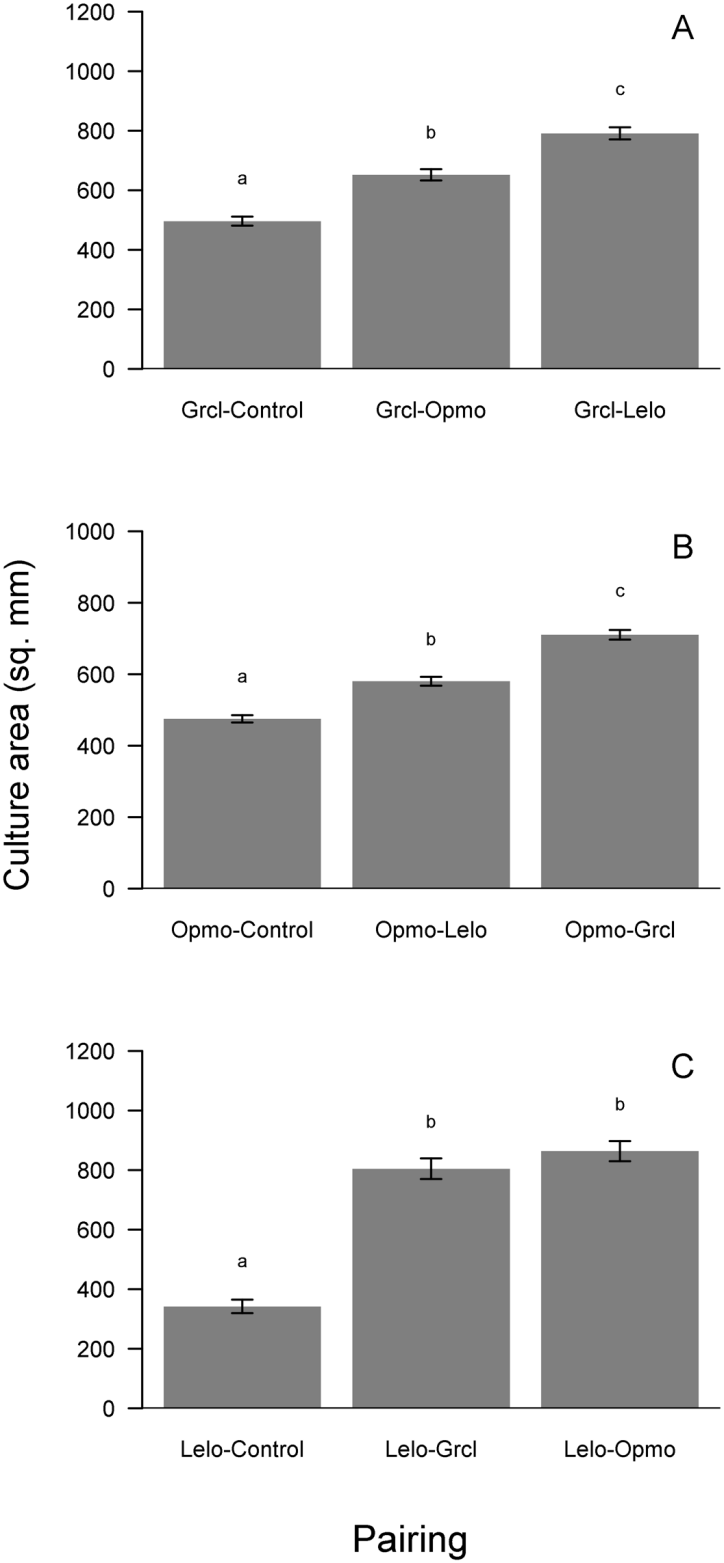
Mean (± s.e.) growth (mm^2^) responses of *Grosmannia clavigera* (Grcl; A), *Ophiostoma montium* (Opmo, B), and *Leptographium longiclavatum* (Lelo; C) on yeast nutrient base agar and exposed to headspace volatiles of other fungi in species-pairing treatments and non-inoculated agar controls. Bars with different letters are statistically different as indicated by Tukey Honest Significant Difference tests.

In a sealed chamber, FVOCs from four-day old cultures of other fungi consistently stimulated the growth of fungi growing on a substrate devoid of usable carbon (i.e., YNB agar). The magnitude of these responses, however, differed by fungus and by FVOC source (i.e., species of origin). *Grosmannia clavigera* growth on YNB agar was significantly (*F*_2, 57_ = 64.93, p<0.001) stimulated by FVOC treatments (Fig 7A). Fungal VOCs from old *O*. *montium* and *L*. *longiclavatum* cultures stimulated *G*. *clavigera* growth by 31% and 59%, respectively (Fig 7a). Similarly, *O*. *montium* growth was significantly (*F*_2, 57_ = 94.21, p<0.001) stimulated by FVOC treatments (Fig 7B). Specifically, FVOCs from *L*. *longiclavatum* and *G*. *clavigera* increased *O*. *montium* growth by 22% and 49%, respectively (Fig 7B). *Leptographium longiclavatum* growth significantly (*F*_2, 57_ = 85.64, p<0.001) responded to FVOC treatments (Fig 7C). Although not different from each other, *L*. *longiclavatum* cultures exposed to *G*. *clavigera* and *O*. *montium* VOCs were 135% and 152% larger, respectively, than controls (Fig 7C).

## Discussion

Carbon-based FVOCs can act as carbon sources to support the growth of co-occurring fungi that share both insect and plant hosts, as demonstrated here with single strains of the MPB symbionts *G*. *clavigera*, *L*. *longiclavatum*, and *O*. *montium*. We showed fungi growing on substrates devoid of usable carbon were larger when exposed to FVOCs of other species. The magnitude of this response differed by fungus and FVOC source (i.e., species of origin). For example, *O*. *montium* cultures were 22% and 49% larger when exposed FVOCs of *L*. *longiclavatum* and *G*. *clavigera*, respectively. However, *L*. *longiclavatum* cultures were 135% and 152% larger when exposed to VOCs of *G*. *clavigera* and *O*. *montium*, respectively. While potentially in part explained by a stimulatory effect of FVOCs, we feel these growth responses are more likely due to use of FVOC-based carbon because growth and biomass accumulation are dependent upon carbon availability often in the form of carbohydrates, which were absent from the experiment substrates. Thus, likely the only environmentally available carbon sources to supply fungal growth and biomass accumulation were FVOCs. To our knowledge, the use of FVOC-based carbon by fungi has not been previously described. However, other ascomycetes can use several industrial volatile hydrocarbons, ketones, and acids as their sole carbon source [40,41]. The amount of usable carbon available however varies by compound as well as species [41]. In regards to biogenic VOCs, some microbes differentially produce enzymes that allow them to derive carbon from plant-based sesquiterpenes [47]. For bark beetle-associated fungi, *G*. *clavigera* possess genes involved in degrading and assimilating carbon from host pine terpenes [48]. Our results indicate that, in addition to pine terpenoids, some bark beetle fungal symbionts can degrade and assimilate carbon from FVOCs. However, their use of such carbon sources may be context-dependent.

Fungal use of FVOC-based carbon may depend upon the availability of substrate-bound carbon. Indeed, we showed that *O*. *montium* exposed to *G*. *clavigera* VOCs produced larger cultures when growing on a substrate without usable carbon, but did not respond (concurrent-growth experiment) or was inhibited (staggered-growth experiment) when growing on carbon-rich substrates. Bark beetle-associated fungi, such as *G*. *clavigera*, can exhibit similar context-dependent degradation of host chemicals, requiring more complex conditions than simple chemical presence [49]. Similarly, fungal use of industrial VOC-based carbon varies with substrate chemistry, such as pH which is an important factor to nutrient acquisition and thus realized availability [41,50]. Infected pine phloem is a heterogeneous nutrient environment, with relatively carbon-rich (e.g., healthy, non-infected phloem) and carbon-poor (e.g., infected phloem, previously colonized by another fungus) patches occurring adjacent to each other [51,52]. For MPB-associated fungi, use of FVOCs may in part explain positive *O*. *montium* performance in *G*. *clavigera*-infected phloem (lesions), which contain less usable carbon than healthy tissue [51,53,54]. However, biological and/or ecological explanations for the growth responses of carbon-limited *G*. *clavigera* and *L*. *longiclavatum* cultures are unclear but may involve supplementation of substrate-bound carbon during host decline. Further, our results may mechanistically explain why *O*. *montium* growth is not nutrient limited in *G*. *clavigera*-infected patches of lodgepole pine phloem [22]. Use of FVOCs as a carbon source may be an adaptive trait facilitating fungal survival in nutrient-poor environments.

Using single strains, we show that communities of bark beetle-associated fungi can be in part regulated by FVOCs acting as semiochemicals in an age- and species-dependent manner, potentially in combination with FVOC-based carbon, to mediate interspecific interactions among component fungi. Volatiles from young *O*. *montium* and *L*. *longiclavatum* cultures in the concurrent-growth experiment stimulated growth, but not conidia production, of *G*. *clavigera*. However in the staggered experiment, FVOCs from older cultures of these same fungi instead inhibited growth and simultaneously stimulated conidia production of *G*. *clavigera*. Further, we showed that FVOCs from a given fungus can selectively (e.g., *O*. *montium* VOCs) or broadly (e.g., *G*. *clavigera* VOCs) inhibit the growth and/or spore production of other fungi. Similarly, an earlier study showed the directionality (inhibition or stimulation) of *G*. *clavigera* responses to *O*. *montium* depended upon fungal proximity and age [54]. Age-related effects of one fungus on another, as shown here and observed by Bleiker and Six [54], are presumably due to variation in FVOC concentrations or composition. Indeed, FVOCs act as semiochemicals in a concentration-dependent manner to inhibit or stimulate fungal growth and reproduction in model systems [26,33–36]. However, a given fungus can respond differently to unique FVOC compositions, such as resulting from different emitter species or environmental conditions [24,26,55]. Further, inhibitory VOCs from competitively dominant fungi can facilitate antagonistic relationships with less competitive, co-occurring fungi, thereby in part affecting fungal community composition [39,55–58]. While additional work using several strains of each species could provide further evidence, our findings indicate that by inhibiting or stimulating fungal growth and conidia production, volatile-mediated interactions are critical determinants of the performance and competition among bark beetle fungal symbionts. Such semiochemical effects may likely drive fungal community dynamics in these systems.

Mountain pine beetle’s symbiotic fungi emit qualitatively and quantitatively different constitutive VOC profiles. Five compounds (acetoin, isobutanol, 2-methyl-1-butanol, 3-methyl-1-butanol, and phenethyl alcohol) were common to VOC profiles of all strains, whereas one compound (*cis*-grandisol) was exclusive to the *G*. *clavigera* strain and three esters (ethyl acetate, isoamyl acetate, and phenethyl acetate) were exclusive to the *L*. *longiclavatum* strain. FVOC concentrations allowed clear discrimination among fungi, which were strongly associated with at least two VOCs. Although *cis*-grandisol has, to our knowledge, never been reported from fungi, other compounds we detected are produced by other fungi. Some of these (2-methyl-1-butanol, 3-methyl-1-butanol, and phenethyl alcohol) are especially common and dominant components of FVOC profiles, as we also observed, here [59–61]. Similarities in dominant FVOCs are indicative of shared or distinct ecological function (e.g., phytopathogen, symbionts, and saprophyte) [59]. Despite this broad clustering, species-level chemotaxonomy show fungi generally emit unique (qualitatively or quantitatively) FVOC profiles and that particular indicator compounds can be used to detect and identify species [62]. Similarities among FVOC profiles of *G*. *clavigera*, *O*. *montium*, and *L*. *longiclavatum* may reflect their shared ecological function as phytopathogens and/or insect symbionts. However, particular indicator-FVOCs may be useful for detecting and identifying these fungi.

Volatiles emitted by MPB fungal symbionts may affect other organisms in this system. Many of the VOCs detected here from the *G*. *clavigera*, *O*. *montium*, and *L*. *longiclavatum* strains are bioactive in other systems. Phenethyl alcohol, 2-methyl-1-butanol, and 3-methyl-1-butanol are common FVOCs that differentially attract many species representing several insect phyla [31]. Phenethyl alcohol in particular is attractive to several bark beetles other than MPB [63,64]. Further, this compound may similarly affect southern pine beetle (*Dendroctonus frontalis*) [65]. This beetle is also more attracted to a semiochemical blend (frontalin-*trans*-verbenol-turpentine) containing phenethyl acetate and isoamyl acetate—esters emitted by its associated yeasts—than to the blend alone [66]. Further isoamyl acetate is toxic to some bacteria and fungi [60]. Lastly, *cis*-grandisol (grandlure I) is an important aggregation pheromone of several *Antonomus* weevil species [67]. Ambrosia beetles are attracted to FVOCs either exclusively from their symbionts or to FVOCs of its symbionts and those from symbionts of other beetles [28]. In this system, odor perception and specificity likely help maintain these symbioses [28]. Bark beetles maintain their symbioses by differentiating their symbiotic fungi from those of other species, exhibiting a high specificity and fidelity modulated by an unknown recognition mechanism [68]. Recognition of fungal symbionts is critical to bark beetle survival [11,12], and our findings suggest that FVOCs may in part be such a recognition signal. Additionally, other microbial associates of bark beetles may be adversely affected by FVOCs.

## Conclusions

While the impact of MPB on North American forests is dependent upon a community of symbiotic fungi, which is comprised of *G*. *clavigera*, *O*. *montium*, and *L*. *longiclavatum* that are critical to beetle attack, we know little of the factors regulating these communities. Using single strains of each species, our study of these MPB-associated symbiotic fungi demonstrates that FVOCs can be important, previously unrecognized factors that act in a context-dependent manner to regulate communities of bark beetle-vectored phytopathogenic fungi by shaping interspecific interactions and fungal survival. While the prevalence and magnitude of these FVOC-mediated interactions in the field is unclear, the semiochemical relationships shown here suggest a colonization/developmental scenario where FVOC-mediated interactions among fungi are relatively minor during the early colonization of pine tissue. As these fungi age, their VOCs exert stronger effects on other community members, such as inhibitory or stimulatory effects on the growth and/or reproduction of later-arriving, younger fungi. Under this scenario, access to FVOC-based carbon may be an evolved trait facilitating resource partitioning, and thus co-existence, in these phytopathogenic fungal communities. Further, we infer that FVOC-mediated effects on conidia production could affect the composition of fungal communities in dispersing MPB, which fill their mycangia with these fungal propagules prior to emergence. Thus, those species producing more conidia would have a greater presence in mycangia of dispersing beetles. Volatiles from MPB’s symbiotic fungi may be useful to species detection and identification, and may act as recognition signals to help maintain bark beetle symbioses. Overall, the findings here provide evidence that FVOCs are important drivers of communities of fungi that naturally co-infect pine hosts attacked by a shared bark-beetle vector.

## Supporting Information

S1 Fig Mean (± s.e.) growth (mm^2^) responses of *Grosmannia clavigera* (Grcl; A), *Ophiostoma montium* (Opmo, B), and *Leptographium longiclavatum* (Lelo; C) on water agar and exposed to headspace volatiles of other fungi in species-pairing treatments and non-inoculated agar controls. Bars with different letters are statistically different as indicated by Tukey Honest Significant Difference tests.
(TIF)
