# Supplementary figures and images for: Fungal Volatiles Can Act as Carbon Sources and Semiochemicals to Mediate Interspecific Interactions Among Bark Beetle-Associated Fungal Symbionts
Source: PLoS One. 2016 Sep 1;11(9):e0162197. doi: 10.1371/journal.pone.0162197 (PMC5008770; doi:10.1371/journal.pone.0162197)

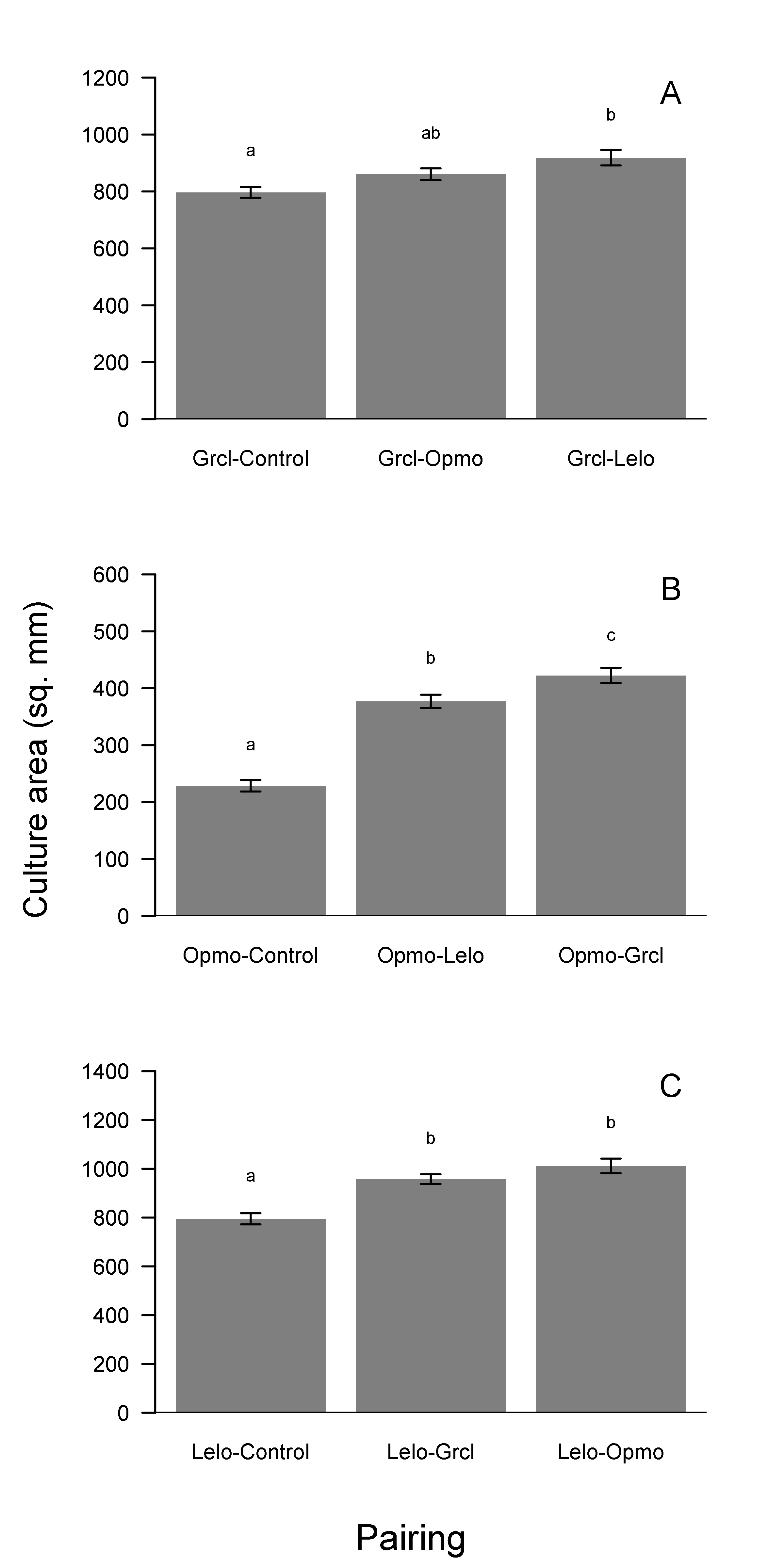

Supplement: S1 Fig — Bars with different letters are statistically different as indicated by Tukey Honest Significant Difference tests. (TIF) [file pone.0162197.s001.tif]
